# Regulation of the Larval Transcriptome of *Diatraea saccharalis* (Lepidoptera: Crambidae) by Maternal and Other Factors of the Parasitoid *Cotesia flavipes* (Hymenoptera: Braconidae)

**DOI:** 10.3389/fphys.2019.01106

**Published:** 2019-09-06

**Authors:** Bruna Laís Merlin, Fernando Luis Cônsoli

**Affiliations:** Insect Interactions Laboratory, Department of Entomology and Acarology, College of Agriculture “Luiz de Queiroz”, University of São Paulo, Piracicaba, Brazil

**Keywords:** calcium signaling regulation, gene expression, host regulation, host–parasitoid interactions, polydnavirus, sustainable pest management

## Abstract

Koinobiont endoparasitoid wasps regulate the host’s physiology to their own benefit during their growth and development, using maternal, immature and/or derived-tissue weaponry. The tools used to subdue the wasps’ hosts interfere directly with host transcription activity. The broad range of host tissues and pathways affected impedes our overall understanding of the host-regulation process during parasitoid development. Next-generation sequencing and *de novo* transcriptomes are helpful approaches to broad questions, including in non-model organisms. In the present study, we used Illumina sequencing to assemble a *de novo* reference transcriptome of the sugarcane borer *Diatraea saccharalis*, to investigate the regulation of host gene expression by the larval endoparasitoid *Cotesia flavipes*. We obtained 174,809,358 reads and assembled 144,116 transcripts, of which 44,325 were putatively identified as lepidopteran genes and represented a substantial number of pathways that are well described in other lepidopteran species. Comparative transcriptome analyses of unparasitized versus parasitized larvae identified 1,432 transcripts of *D. saccharalis* that were up-regulated under parasitization by *C. flavipes*, while 1,027 transcripts were down-regulated. Comparison of the transcriptomes of unparasitized and pseudoparasitized *D. saccharalis* larvae led to the identification of 1,253 up-regulated transcripts and 972 down-regulated transcripts in the pseudoparasitized larvae. Analysis of the differentially expressed transcripts showed that *C. flavipes* regulated several pathways, including the Ca^+2^ transduction signaling pathway, glycolysis/gluconeogenesis, chitin metabolism, and hormone biosynthesis and degradation, as well as the immune system, allowing us to identify key target genes involved in the metabolism and development of *D. saccharalis*.

## Introduction

Parasitoids have developed a variety of adaptive processes to colonize and successfully develop in a range of host stages (Godfray, 1994). The processes that parasitoids employ vary with the parasitoids’ developmental strategies, but all parasitoids manipulate the host’s physiology and metabolism, in a process known as host regulation (Vinson and Iwantsch, 1980). Host regulation may rely on passive and/or active mechanisms (Schmidt et al., 2001). Parasitoids use passive mechanisms to elude host immune cells by hiding their eggs in tissues to which host hemocytes have limited access, or by covering their eggs with immunoevasive proteins produced in the wasp ovaries (Schmidt et al., 2001; Dorémus et al., 2013a). Mechanisms of active regulation are stimulated by maternal factors (ovarian proteins, venom, and viral particles) injected during oviposition, or by factors that parasitic larvae and/or teratocytes release during growth. Active mechanisms alter the host physiology in such a way that the host becomes a suitable environment to sustain parasitoid growth and development (Yu et al., 2007; Asgari and Rivers, 2011; Cônsoli and Vinson, 2012; Dorémus et al., 2013a; Strand and Burke, 2013; Strand, 2014). Strategies for host regulation by koinobionts can be quite variable. The venom may play no role in evading the host immune system in *Hyposoter didymator* (Thunberg) (Hymenoptera: Ichneumonidae), whereas it is pivotal in *Cotesia chilonis* (Munakata) (Hymenoptera: Braconidae) (Dorémus et al., 2013b; Teng et al., 2016).

Members of some subfamilies of Braconidae and Ichneumonidae have mutualistic associations with polydnaviruses (PDV). In the wasp, two forms of PDV DNAs are recognized: viral and insect. DNA sequences in the former category have a viral origin, but are not packaged. Instead, DNA sequences of non-viral origin are packaged for delivery to the parasitized host. The virus particles are released into the calyx lumen, to be injected with the wasp eggs at parasitization. DNA packaging occurs during viral morphogenesis, a process that is restricted to cells of the ovarian calyx of female wasps (Kroemer and Webb, 2004; Burke and Strand, 2012). PDV infects a range of cells of the host, affecting the host’s cellular machinery to regulate several aspects of the host physiology (Strand, 2008). The expression of viral genes leads to regulation of ecdysteroidogenesis and of ecdysteroid secretion by prothoracic glands in the host during parasitization (Pruijssers et al., 2009; Kim et al., 2013; Ignesti et al., 2018). Proteins encoded by genes of the encapsidated form are also required to regulate the host immune response. The humoral immune response is affected by regulation of translation of proteins needed to activate the phenoloxidase cascade (Beck and Strand, 2007; Prasad et al., 2014), and the cellular immune response is affected through the disruption of the cellular cytoskeleton, and the hemocyte attachment and spreading behavior (Pruijssers and Strand, 2007; Kumar and Kim, 2016). PDV also regulates the host metabolism by affecting nutrient uptake and allocation (Thompson, 2001; Bottjen, 2011; Di Lelio et al., 2014; Kumar et al., 2016). Recent data showed that host regulation by PDV also influences the herbivore’s interaction with the host plant, lowering the capacity of parasitized larvae to elicit the plant’s immune response, by reducing the availability of salivary glucose oxidase (Tan et al., 2018).

Research to identify the bioactive molecules that parasitoids use to regulate their hosts and expand our understanding of the regulation of host gene expression has intensified, with next-generation sequencing techniques and bioinformatic tools supporting systemic analyses (Fang et al., 2010; Etebari et al., 2011; Provost et al., 2011; Laurino et al., 2016). Genomic analyses are elucidating the organization of the integrated and encapsidated forms of the PDV genome (Chevignon et al., 2014), and transcriptome analyses are depicting the profile of viral and host gene expression in parasitized hosts (Bitra et al., 2011; Provost et al., 2011; Dorémus et al., 2014; Ali et al., 2015). Integrative analyses have identified bioactive molecules present in maternal factors injected by the parasitoid (Laurino et al., 2016; Liu et al., 2017).

*Diatraea saccharalis* (F.) (Lepidoptera: Crambidae) is the major pest of sugarcane in Brazil causing direct and indirect damage. Losses in sugar and ethanol production due to insect damage to sugarcane can be as high as 10%, resulting in an economic loss of nearly US$ 4,6 billion/year (Oliveira et al., 2014). The larval boring behavior also limits the use of chemical strategies for the control of this pest, and the use of biological control through macrophages has been the best strategy to manage this pest (Botelho and Macedo, 2002). Among all natural enemies used to control the sugarcane borer, *Cotesia flavipes* (Cameron) (Hymenoptera: Braconidae) is the only parasitoid that is currently mass-produced to target the larval stage of this pest. *Cotesia flavipes* is exotic to Brazil. It is a common larval endoparasitoid of several species of Crambidae and other borers in Southeast Asia and Australia. *Cotesia flavipes* was introduced early in the 1970s from Trinidad, where it has been introduced earlier from Mauritius and India (Botelho and Macedo, 2002). Biological control with *C. flavipes* requires the constant release of adult wasps in the field, which keeps the infestation levels of *D. saccharalis* close to 2–3% (Botelho and Macedo, 2002). The successful exploitation of *D. saccharalis* by *C. flavipes* is partially due to the demonstrated capacity of this parasitoid to regulate its host physiology. *Cotesia flavipes* has been demonstrated to alter the host metabolism by affecting the availability of sugars and proteins circulating in the hemolymph and stored in the fat body tissues (Salvador and Cônsoli, 2008), and by influencing food consumption and utilization by parasitized larvae (Rossi et al., 2014). *Cotesia flavipes* also affects the host cellular and humoral immune response by affecting the total hemocyte population and phenoloxidase activity (Mahmoud et al., 2011, 2012). The suppression of the immune system of *D. saccharalis* by *C. flavipes* is highly effective, but the efficiency in escaping host immune response (egg encapsulation) is reduced in *Diatraea grandiosella* (Dyar) (Lepidoptera: Crambidae) (Alleyne and Wiedenmann, 2001) and completely ineffective in avoiding encapsulation by larvae of *Ostrinia nubilalis* (Walker) (Lepidoptera: Pyralidae) (Alleyne and Wiedenmann, 2001) and *Manduca sexta* (L.) (Lepidoptera: Sphingidae) (Rodríguez-Pérez et al., 2005). Like other species in the *flavipes* complex, *C. flavipes* is also associated with a polydnavirus (Cônsoli and Kitajima, 2006). PDV associated with species in the *flavipes* complex as well as bracoviruses and ichnoviruses associated with other wasp species are known to have a key role in the immunosuppression of parasitized hosts (Ye et al., 2018). The failure of *C. flavipes* to immunosuppress *O. nubilalis* and *M. sexta* suggests a narrow physiological range for the *C. flavipes* PDV-produced imunnosuppressants (Alleyne and Wiedenmann, 2001; Rodríguez-Pérez et al., 2005).

The broad array of functional targets that parasitic wasps are able to regulate and the diversity of bioactive molecules that they produce to successfully control several attributes of the host biology and physiology have great potential for the development of sustainable biotechnologies for pest management (Beckage and Gelman, 2004). Successful exploitation of the host regulation process as a source of new targets and/or bioactive molecules relies on identification and understanding of the functional processes affected during this process. We investigated the process of regulation of the gene expression of the sugarcane borer *D. saccharalis* parasitized by the larval endoparasitoid *C. flavipes*. Our purpose was to identify target genes and the pathways of the host that are regulated by maternal factors injected by female wasps during parasitization, or by factors that are released by the developing immature parasitoids, using comparative transcriptome analyses of control, parasitized and pseudoparasitized larvae, based on a *de novo* transcriptome of *D. saccharalis* larvae.

We provide the first larval transcriptome of the sugarcane borer *D. saccharalis* and an in depth analysis of the gene expression regulation of sugarcane borer larvae by its endoparasitoid *C. flavipes*. Our data add to the existing literature of host regulation by parasitoids through the investigation of an undescribed system, and the investigation of the regulation of the host gene expression in natural and pseudoparasitized larvae. We expect our data will contribute to the discovery of target genes and/or pathways of the sugarcane borer amenable to manipulation that could be further exploited in the development of alternative strategies for pest control.

## Materials and Methods

### Rearing of *D. saccharalis* and *C. flavipes*

A stock colony of *D. saccharalis* was reared on an artificial diet based on soy flour and wheat germ (King et al., 1985) under controlled laboratory conditions (25 ± 1°C; 60 ± 10% RH; photophase of 14 h). A stock colony of *C. flavipes* was maintained using fourth and fifth instars of *D. saccharalis* as hosts for parasitization. The stock colony of *D. saccharalis* was obtained from field collections in the region of Piracicaba, state of São Paulo, and has been maintained in culture for the last 10 years without introduction of field-collected insects. *Cotesia flavipes* was obtained from sugarcane mills insectaries, and it belongs to the original colony that was introduced from Trinidad into Brazil (Botelho and Macedo, 2002). The host larvae were offered individually to female wasps for a single sting. Once female wasps stung the larvae, larvae were individually transferred to rearing containers containing new artificial diet, until the parasitoid larvae exited from the host larvae. After cocoon formation, the masses of cocoons were collected and transferred to clean plastic dishes until the wasps emerged.

### Irradiation of *C. flavipes* Cocoons

In order to obtain females that would lay inviable, sterile eggs for the pseudoparasitization experiments, cocoons containing late-stage pupae (7–8 days old) of the parasitoid *C. flavipes* were collected and subjected to ^60^Co gamma irradiation at 75 Gy in a GammaCell 220 irradiator (MDS Nordion, Ottawa, ON, Canada) at the Laboratory of Radiology and Environment – Center of Nuclear Energy in Agriculture (CENA/USP), to sterilize the adults. Previous data from our laboratory showed the irradiation dosage applied led to the complete sterilization of the developing adults, with no harm to their successful emergence and host parasitization (Lopes, 2008). After irradiation, cocoons were maintained in controlled conditions until adults’ emergence and parasitization.

### Parasitization of *D. saccharalis* Larvae by *C. flavipes*

Newly molted fifth-instar of *D. saccharalis* were divided in three subgroups: (i) control, larvae that were not parasitized; (ii) parasitized, larvae that were individually parasitized by naïve, fed and mated *C. flavipes* females, exposing the hosts to virulence factors from the calyx fluids, PDV particles, venom, and larval and teratocyte secretions of the parasitoid; and (iii) pseudoparasitized, larvae that were individually parasitized by gamma-irradiated sterile *C. flavipes* females, exposing the hosts only to virulence factors from the calyx fluids, PDV particles, and venom of the wasp. Larvae that were subjected to parasitization or pseudoparasitization were stung only once by female wasps. Subsamples of parasitized and pseudoparasitized larvae were removed for later dissection to confirm the level of successful parasitization (parasitized) or female sterilization (pseudoparasitized) by inspection of the parasitoid larvae. All larvae were placed in individual tubes containing artificial diet and maintained in controlled laboratory conditions as described above, to allow them to develop.

### Tissue Collection and RNA Extraction of *D. saccharalis*

Larvae of *D. saccharalis* parasitized and pseudoparasitized by *C. flavipes* and unparasitized control larvae were sampled 1, 3, 5, and 7 days after parasitization and immediately placed in RNA*later* stabilization solution (Thermo Fisher Scientific), and then stored at −80°C until RNA extraction. Each sampling period had three biological replicates, each replicate consisting of a pool of five larvae. Forced by a sudden limitation in funds, the total RNA later obtained from each sampling period had to be pooled, and each pooled sample represented one sample with a mixture of larvae sampled 1, 3, 5, and 7 days after parasitization.

Larval tissues were disrupted using a TissueLyser II LT^TM^ (Qiagen), following the manufacturer’s recommendations. The processed tissues were transferred to 1.5 mL microtubes containing 1 mL TrizolTM reagent (Invitrogen). Samples were homogenized manually, centrifuged (12,000 *g* × 10 min × 4°C), and the upper layer mixed with 200 μL chloroform. Samples were homogenized by inversion, incubated at room temperature for 3 min and centrifuged (12,000 *g* × 15 min × 4°C). The aqueous layer was collected, added to 500 μL isopropanol, incubated for 10 min and centrifuged (12,000 *g* × 10 min × 4°C). The pellet was washed with 75% ethanol and centrifuged (7,500 *g* × 5 min × 4°C) twice. The samples were allowed to dry at room temperature and the pelleted RNA was recovered in 30 μL RNAse-free water and incubated at 60°C for 15 min.

Total RNA was quantified using the EpochTM Microplate Spectrophotometer (Fisher Scientific). After quantification, samples collected at 1, 3, 5, and 7 days after parasitization were used in the preparation of an equimolar mixture for each biological replicate of the three treatments: (i) control, (ii) parasitized, and (iii) pseudoparasitized. Each biological replicate represented a pool of 20 larvae sampled at 1, 3, 5, and 7 days after parasitization. Each treatment was represented by three biological replicates, totaling 60 larvae for each treatment.

### cDNA Sequencing

Differentially expressed genes of *D. saccharalis* larvae parasitized or pseudoparasitized by *C. flavipes* were identified by sequencing cDNA libraries using the Illumina© HiScanSQ platform, available at the Multiusers Center of Agricultural Biotechnology at the Department of Animal Sciences, ESALQ/USP.

RNA integrity was confirmed using the Agilent Bioanalyzer 1000 (Agilent Technologies). RNA samples were prepared for sequencing using the cDNA TruSeq RNA Library Prep Kit (Illumina©) following a paired-end (2 × 100 bp) strategy. Briefly, mRNA was purified from total RNA using oligo(dT) magnetic beads and fragmented into sequences of 200 nucleotides. Fragmented mRNA was ligated to random primers, followed by the reverse transcription reaction for cDNA library construction. The cDNA produced was end-repaired and purified using magnetic beads and ethanol washing. After the end repair, adenosine was added at the 3′ end of each cDNA fragment to guide the ligation of specific adapters. Adapters consisted of primers for transcription primers and a specific index to code each sample. Samples were enriched with limited-cycle PCR and analyzed to confirm the success of sample preparation.

### *De novo* Transcriptome Assembly

Reads obtained from sequencing the cDNA libraries of the control, parasitized and pseudoparasitized larvae of *D. saccharalis* were visualized in FastQC software to verify the quality (Andrews, 2010). Reads were then analyzed using Trimmomatic-0.36 (Bolger et al., 2014) for clipping Illumina adapters and quality filtering by trimming the leading (LEADING:3) and trailing (TRAILING:3) nucleotides until the quality was higher than 3, and then using a sliding window of 4 nucleotides and trimming when scores were lower than 22 (SLIDINGWINDOW 4:22). Remaining reads shorter than 25 nucleotides were also excluded.

Reads obtained from all replicates of the three treatments (control, parasitized, and pseudoparasitized) were used to assemble a *de novo* transcriptome, using the pipeline available in the Trinity-v.2.4.0 software (Haas et al., 2013). Both paired and unpaired trimmed and quality filtered reads were used to assemble a *de novo* transcriptome that was used as the reference transcriptome for the RNASeq experiments. Assemblage was obtained using normalization of the reads coverage (<50), and the minimum contig size selected was 200 nucleotides.

The transcripts obtained were functionally annotated using the BlastX algorithm for putative identification of homologous sequences, with an *e*-value cut-off < 10^–3^. Annotated sequences were cured and grouped into categories according to their function, using Blast2Go^®^ (Conesa et al., 2005), with an *e*-value cut-off < 10^–6^ and EggNOG-mapper 4.5.1 (Huerta-Cepas et al., 2017). Transcripts putatively identified as belonging to lepidopteran genes were checked against the KEGG database (Kyoto Encyclopedia of Genes and Genomes) (Kanehisa and Goto, 2000) to verify the metabolic pathways represented in the obtained *de novo* transcriptome.

### Differential Gene Expression Analyses

The differential gene expression among the control and larvae of *D. saccharalis* parasitized and pseudoparasitized by *C. flavipes* was analyzed using the software RSEM (Li and Dewey, 2011). Reads from each library were counted against the *de novo* transcriptome and counts were normalized as transcripts per million reads (TPM). TPM values for each sample sequenced were used to calculate fold-change ratios for comparative analyses of the gene expression of control (NP) versus parasitized (P), and control versus pseudoparasitized (PP) larvae. Analyses were carried out using the statistical package EdgeR (Robinson et al., 2010), and the values of fold change obtained were corrected with the False Discovery Rate (FDR) method. Only transcripts that showed *p* < 0.05 and log fold change > | 2| between treatments were considered differentially expressed. Differentially expressed transcripts were grouped according to the physiological processes in which they are involved.

## Results

### *De novo* Transcriptome Assembly

Reads obtained from the control and larvae of *D. saccharalis* parasitized and pseudoparasitized by *C. flavipes* had a mean quality score >32 for each nucleotide position, with less than 2% being filtered (Supplementary Table S1). Quality filtering and trimming yielded 174,809,358 reads, which resulted in the assembly of 144,116 transcripts (Supplementary Table S2). Annotation of the *de novo* assembly allowed a putative identification of 84,678 transcripts, of which 14,177 sequences were allocated to different gene ontology categories.

**FIGURE 1:**
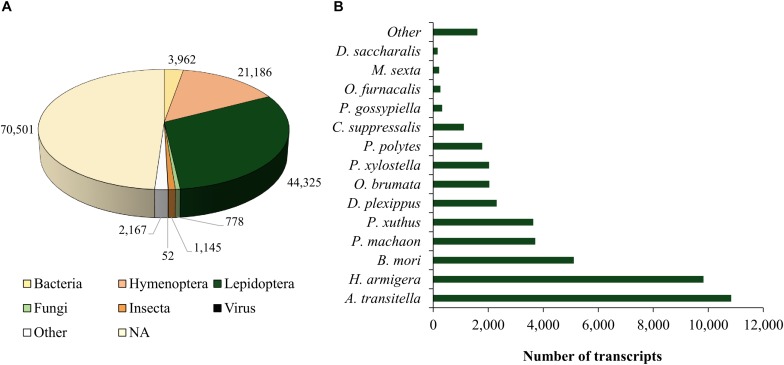
Distribution of alignments and transcripts of *de novo* assembly of control larvae of *Diatraea saccharalis* parasitized and pseudoparasitized by *Cotesia flavipes* after search for similarity in NCBI databank; **(A)** Groups represented by *de novo* assembly; **(B)** Lepidoptera species obtained in the annotation of transcripts of *D. saccharalis: Manduca sexta; Ostrinia furnacalis; Pectinophora gossypiella; Chilo suppressalis; Papilio polistes; Ophemerata brunata; Danaus plexippus; Papilio xuthus; Papilio machaon; Bombyx mori; Helicoverpa armigera; Amyelois transitella*.

**FIGURE 2:**
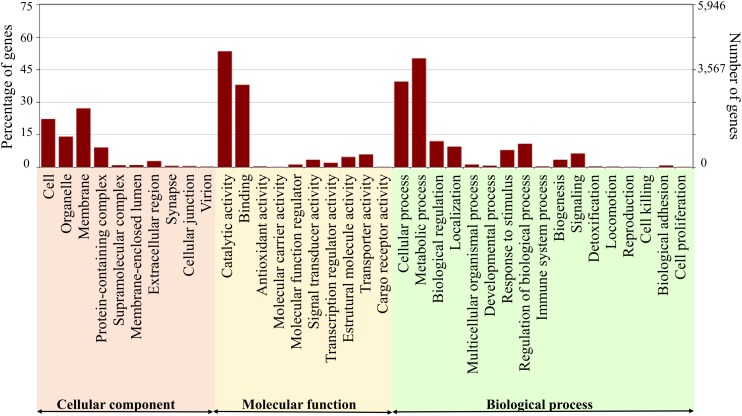
Distribution of functionally annotated transcripts of *Diatraea saccharalis* in gene ontologies.

One-third of the annotated transcripts were similar to genes belonging to Lepidoptera and approximately 15% to Hymenoptera (Figure 1A). Of the 44,325 transcripts expressed by *D. saccharalis* that were similar to Lepidoptera sequences, 26% were similar to *Amyelois transitella* (Walker) (Lepidoptera: Crambidae), 22% to *Helicoverpa armigera* (Hübner) (Lepidoptera: Noctuidae), and 11.5% to *Bombyx mori* (L.) (Lepidoptera: Bombycidae) sequences (Figure 1B). Annotation of 7,928 transcripts resulted in their grouping in categories of cellular component (10), molecular function (10), and biological process (17) (Figure 2). The enzyme codes generated from the annotation of the transcriptome of *D. saccharalis* led to the identification of 7,006 transcripts represented in the metabolic pathways using KEGG (Kanehisa and Goto, 2000), confirming the quality and representativeness of the *de novo* assembly obtained (Supplementary Figure S1).

### Differential Gene Expression Analyses

**FIGURE 3:**
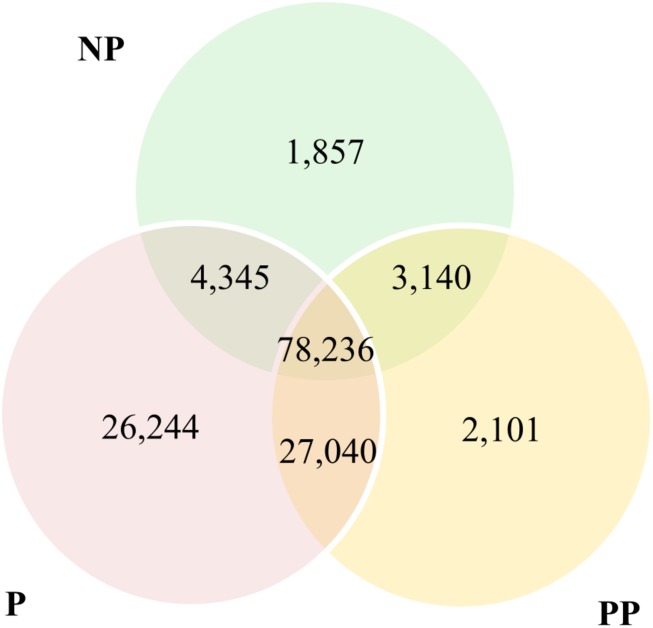
Venn diagram of transcripts of the *de novo* assembly of *Diatraea saccharalis* larvae expressed in control (NP) and parasitized (P) and pseudoparasitized (PP) larvae by *Cotesia flavipes.*

Unparasitized, parasitized and pseudoparasitized larvae of *D. saccharalis* by *C. flavipes* shared more than 50% of the transcripts from the *de novo* assembly (Figure 3). *D. saccharalis* larvae non-parasitized and pseudoparasitized by *C. flavipes* had about 1% exclusive transcripts, while parasitized larvae had more than 20% exclusive transcripts (Figure 3).

Differential expression gene analysis indicated that a total of 2,459 transcripts of *D. saccharalis* larvae were differentially expressed when parasitized by *C. flavipes*. The majority of the differentially expressed transcripts (1,764) were down- or up-regulated, 625 were expressed exclusively in parasitized hosts, and 70 were completely inhibited by parasitization. A smaller number of differentially expressed genes (2,225) were identified in larvae of *D. saccharalis* pseudoparasitized by *C. flavipes*. The number of transcripts unique to pseudoparasitized larvae (643) was close to the number of unique transcripts in parasitized larvae (625), but 97 transcripts were completely inhibited in pseudoparasitized larvae compared with the 70 transcripts in parasitized larvae. A total of 1,485 transcripts were up- or down-regulated in pseudoparasitized larvae. Although the number of differentially expressed genes differed between parasitized and pseudoparasitized larvae, we found that both sets of differentially expressed genes represented the same functional groups.

**FIGURE 4:**
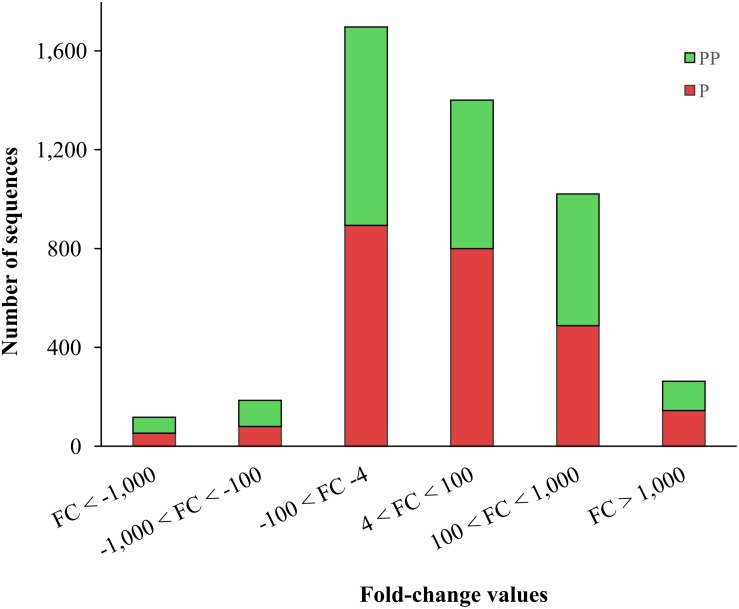
Fold change of differentially expressed genes of *Diatraea saccharalis* larvae parasitized (P) or pseudoparasitized (PP) by *Cotesia flavipes.*

The 1,027 transcripts down-regulated in parasitized larvae differed by fold changes from −4 to more than −10,000-fold, with the majority differing in expression by fold-change values between −4 and −100-fold (Figure 4). Fold-change values of the 1,432 up-regulated transcripts in parasitized larvae ranged from 4 to 1,000 times. Fold-change values for differentially expressed genes in pseudoparasitized larvae were similar to the values observed for parasitized larvae. Values for fold change for the 972 sequences of *D. saccharalis* that were down-regulated in pseudoparasitized larvae ranged from −4 to −100, while the fold changes for the 1,253 up-regulated sequences were mainly between 4 and 1,000-fold (Figure 4).

A serine protease inhibitory protein (IIL_32799_c0_g1_i4: logFC = 7) and a defensin precursor (IIL_25072_c0_g1_i1: logFC = 12.74) were included in the group of transcritps with the highest fold-change differences after parasitization, while cuticular proteins (IIL_26787_c0_g1_i3: logFC = –10.12; IIL_31999_c0_g2_i4: logFC = –10.10) were the most strongly regulated in parasitized larvae. In pseudoparasitized larvae, genes of the immune humoral response, i.e., trypsin and Toll proteins (IIL_33635_c0_g1_i23: logFC = 6.84; IIL_34261_c0_g1_i7: logFC = 7.36), were among the most strongly regulated. Expression of genes coding for cuticular proteins was also strongly inhibited in pseudoparasitized larvae (IIL_31999_c0_g2_i4: logFC = –14.23; IIL_28404_c0_g3_i1: logFC = –11.54; IIL_21160_c0_g1_i1: logFC = –10.57; IIL_23374_c0_g1_i1: logFC = –9.95).

### Regulation of Cell Cycle and Apoptosis

The expression of genes involved in cell cycle regulation was affected in parasitized and pseudoparasitized larvae, but a much higher number of differentially expressed transcripts was detected in parasitized than in pseudoparasitized larvae. DNA duplication and cell cycle progression were affected by the down-regulation of cyclin-dependent kinases in both conditions (IIL_22184_c0_g1_i1: cyclin-dependent kinase 20, logFC = –2.91 in P; logFC = −3.39 in PP; IIL_27801_c0_g1_i3: cyclin-dependent kinase 10, logFC = −2.87 in P; −2.29 in PP; IIL_29239_c4_g1_i2: cyclin-dependent kinase 2, logFC = –4.01 in P).

Larval parasitization by *C. flavipes* also led to the down-regulation of inducers of cell apoptosis in parasitized and pseudoparasitized *D. saccharalis*, such as the apoptosis-inducing factor 1 (IIL_32688_c2_g2_i1: logFC = −2.74 in P; IIL_32688_c2_g2_i3: logFC = –2.06 in P; IIL_32688_c2_g2_i6: logFC = –2.55 in P; IIL_32688_c2_g2_i7: logFC = −3.85 in P; −3.87 in PP) and programed cell death 4 (IIL_3726_c0_g2_i1: logFC = –9.85 in P). However, the apoptotic chromatin condensation inducer in the nucleus-like and the death-associated kinase related isoform X2 were up-regulated (IIL_32093_c0_g1_i2: logFC = 8.84 in P, logFC = 8.78 in PP). Caspase 1 and 4 were also up-regulated (IIL_30089_c3_g1_i4: logFC = 7.65 in P; logFC = 8.68 in PP; IIL_31764_c1_g1_i11: logFC = 8.42 in P; IIL_31764_c1_g1_i12: logFC = 3.05 in P; logFC = 2.78 in PP). Enzymes involved in regulation of transduction signaling pathways that result in cellular apoptosis and degradation of reactive oxygen species, and detoxification (ABC transporters, glutathione S-transferase and cytochrome P450) were up-regulated in both parasitized and pseudoparasitized *D. saccharalis* larvae.

### Regulation of Ribosomal Genes and Host Transcription/Translation Factors

**FIGURE 5:**
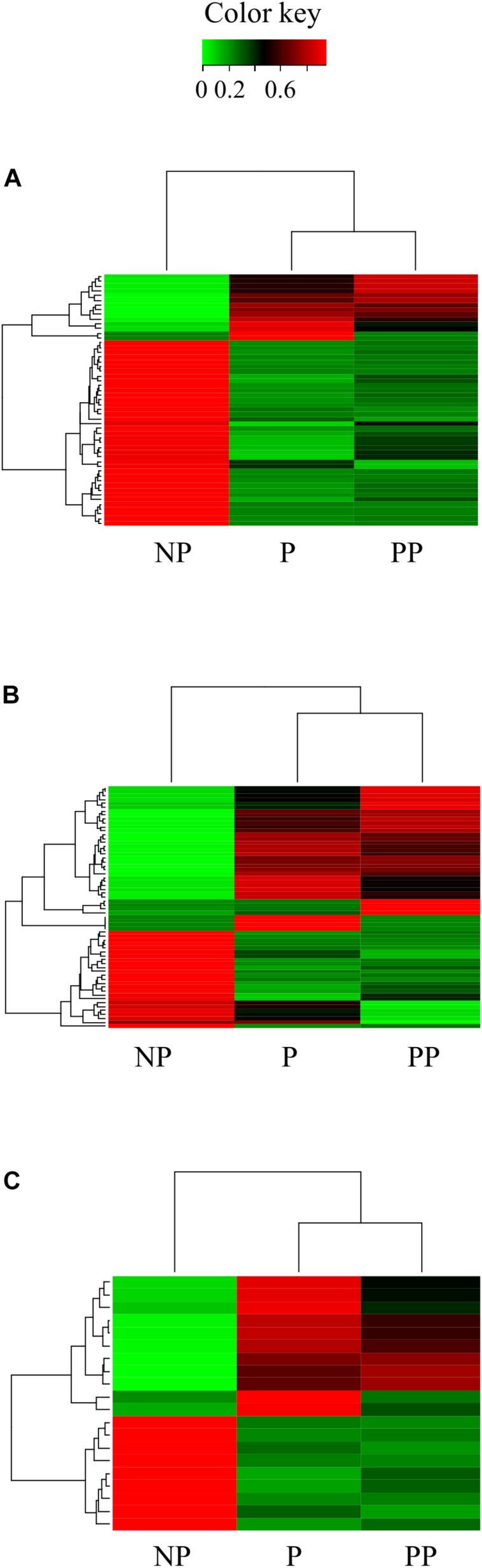
Heatmap of sequences of **(A)** ribosomal proteins, **(B)** transcription factors, and **(C)** translation factors regulated in larvae *Diatraea saccharalis* parasitized and pseudoparasitized by *Cotesia flavipes.*

Most of the ribosomal-protein genes were found to be regulated in parasitized and pseudoparasitized larvae. Thirty-seven ribosomal genes were down-regulated and 12 were up-regulated in parasitized larvae. A smaller number of ribosomal protein genes were affected in pseudoparasitized larvae, with 26 down-regulated and 10 up-regulated genes. Mitochondrial 28S ribosomal, 39S ribosomal L13, and 40S ribosomal L subunits were down-regulated in both larvae attacked by *C. flavipes* (Figure 5A).

Host transcription factors were targeted for regulation in both parasitized and pseudoparasitized larvae (Figures 5B,C). We identified a total of 61 transcription factors that were differentially expressed. Twenty-two were equally regulated in parasitized and pseudoparasitized larvae. Levels of expression of another 24 transcription factors were altered exclusively in parasitized larvae, e.g., the nuclear transcription factor E75B (IIL_29251_c0_g1_i5: logFC = −2.10) and POU class transcription factor (IIL_24864_c0_g1_i1: logFC = −2.54; IIL_24864_c0_g1_i2: logFC = −2.02). However, the histone deacelylase 6 isoform X2 (IIL_24484_c0_g1_i2: logFC = –3.22) and 15 other gene expression regulators were altered only in pseudoparasitized larvae (Figure 5B). *Cotesia flavipes* regulation of host gene expression also targeted the transcription repressor factor ‘transcriptional repressor YY1-like isoform X1’ (transcript IIL_30586_c0_g1_i2, logFC = 9 in P; logFC = 9.85 in PP). Post-transcriptional regulation of the host was also observed in larvae of *D. saccharalis* parasitized or pseudoparasitized by *C. flavipes*, with up-regulation of the translational repressor factor ‘eukaryotic translation initiation factor 4E-binding 2′ (4E-BPs) (IIL_30132_c1_g1_i4: logFC = 2.40 in P, logFC = 2.61 in PP; IIL_31514_c0_g1_i13: logFC = 4.63 in P). 4E-BPs bind to eukaryotic translation initiation factors (eIFs), preventing translation. eIFs are proteins that direct ribosomes to the 5′-cap of mature mRNA. Besides the up-regulation of repressors of eIFs, larvae of *D. saccharalis* that were parasitized or pseudoparasitized by *C. flavipes* also had the expression levels of several eIFs (eukaryotic translation initiation factor 2, 3F and 5B) down-regulated (Figure 5C).

### Hormonal Regulation

**FIGURE 6:**
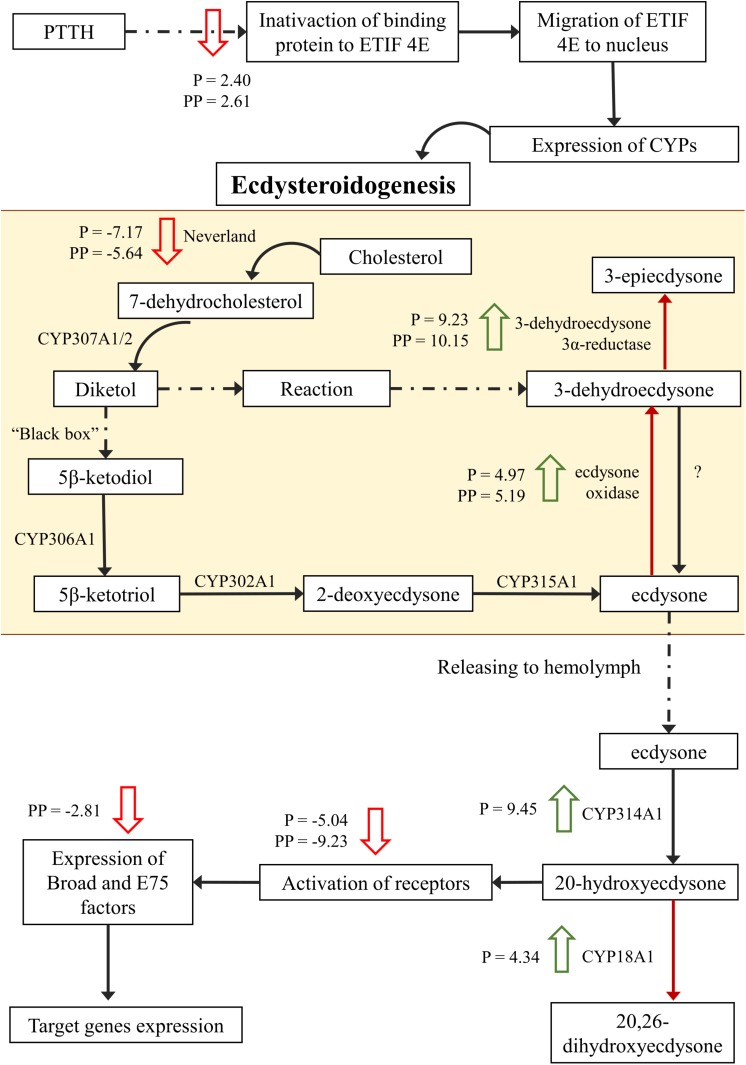
Simplified pathways for ecdysteroid synthesis and release in prothoracic glands. Dashed arrows indicate different reactions and red arrows indicate ecdysteroid inactivation. Large green arrows indicate superexpression of enzymes in *Diatraea saccharalis* parasitized (P) and/or pseudoparasitized (PP) by *Cotesia flavipes*. Large red arrows indicate inhibition of expression in parasitized and/or pseudoparasitized larvae. CYP, cytochrome P450 enzymes; ETIF, eukaryotic translation initiation factor; PPTH, prothoracicotropic hormone.

Regulation of target pathways by *C. flavipes* was observed not only through the regulation of transcription factors, but also of other genes belonging to the pathway. The host hormone metabolism was affected by regulation of the E75B transcription factor (IIL_29251_c0_g1_i5) and of another 28 transcripts in parasitized and 19 transcripts in pseudoparasitized larvae involved in hormone metabolism. Parasitized and pseudoparasitized larvae shared 15 differentially expressed genes that are involved in hormone metabolism (Table 1). Positive regulation of farnesyl diphosphate synthases 1 and 2 (IIL_32117_c1_g1_i1; IIL_34096_c0_g1_i1) and cytochrome P450 18A1 (IIL_31541_c0_g3_i2; IL_31541_c0_g3_i6), enzymes involved in isoprenoid biosynthesis and in the hydroxylation of ecdysone to 20-hydroxyecdysone respectively, was observed in parasitized larvae. Nevertheless, up-regulation of enzymes involved in the degradation of JH and ecdysteroids was also detected in both the parasitized and pseudoparasitized conditions (Table 1 and Figure 6). Additionally, down-regulation of hormone receptors occurred in parasitized and pseudoparasitized larvae (Table 1), but ecdysone-inducible translation factors, such as 74EF and E75, were inhibited only in pseudoparasitized larvae (Table 1).

**TABLE 1:** Putative identification and fold-change values of transcripts related to the endocrine system of larvae of *Diatraea saccharalis* parasitized (P) and pseudoparasitized (PP) by *Cotesia flavipes.*

**Putative identification**	**Transcripts**	LogFC^*1^ in P	FDR^*2^	**LogFC in PP**	**FDR**
3-dehydroecdysone 3α-reductase	IIL_29686_c0_g1_i1	9.23	3.14 × 10^–5^	10.15	2.28 × 10^–6^
Parathyroid hormone-responsive B1	IIL_33372_c0_g2_i2	–3.21	1.77 × 10^–3^	–3.01	1.70 × 10^–2^
Cytochrome P450 monooxygenase CYP18A1	IIL_31541_c0_g3_i2	2.92	2.58 × 10^–4^	−	–
	IIL_31541_c0_g3_i6	4.34	4.16 × 10^–3^	−	–
Nuclear receptor coactivator 5 isoform X1	IIL_33867_c3_g1_i3	8.00	1.86 × 10^–4^	−	–
Rieske-domain Neverland	IIL_25348_c0_g1_i1	–7.17	2.95 × 10^–5^	–5.64	4.27 × 10^–3^
Ecdysone 20-monooxygenase isoform X2 (CYP314A1)	IIL_33026_c0_g13_i2	8.78	2.68 × 10^–5^	−	–
	IIL_33026_c0_g13_i3	9.45	1.21 × 10^–5^	−	–
Ecdysone oxidase	IIL_33220_c0_g1_i8	2.35	1.58 × 10^–2^	−	–
	IIL_34436_c2_g2_i1	4.97	1.62 × 10^–3^	5.19	9.92 × 10^–3^
Juvenile hormone epoxide hydrolase	IIL_33751_c1_g1_i1	2.19	6.10 × 10^–3^	2.20	1.50 × 10^–2^
	IIL_33751_c1_g1_i5	2.21	8.98 × 10^–3^	2.72	3.32 × 10^–2^
	IIL_33290_c1_g1_i4	2.81	7.71 × 10^–3^	2.88	2.18 × 10^–2^
	IIL_33290_c1_g1_i7	2.88	5.23 × 10^–3^	2.97	1.47 × 10^–2^
	IIL_33290_c1_g1_i1	5.55	2.50 × 10^–3^	5.08	1.22 × 10^–2^
Juvenile hormone esterase-like	IIL_29119_c0_g1_i2	2.77	6.23 × 10^–3^	2.71	3.32 × 10^–2^
Farnesyl diphosphate synthase 1	IIL_32117_c1_g1_i1	8.50	3.80 × 10^–5^	−	–
Farnesyl diphosphate synthase 2	IIL_34096_c0_g1_i1	7.76	4.41 × 10^–4^	−	–
Ecdysone-induced 74EF isoform X1	IIL_33545_c3_g1_i3	−	–	–3.26	3.81 × 10^–2^
Ecdysone-inducible E75 isoform X1	IIL_29251_c0_g1_i2	−	–	–2.81	3.85 × 10^–2^
Phosphomevalonate kinase isoform X1	IIL_33071_c1_g4_i5	−	–	4.08	4.36 × 10^–2^
Geranylgeranyltransferase type-1 subunit α	IIL_33557_c0_g1_i2	5.08	3.45 × 10^–4^	5.08	2.46 × 10^–3^
Krueppel homolog 2	IIL_30739_c1_g3_i2	4.42	4.28 × 10^–3^	−	–
Prostamide/prostaglandin F synthase-like	IIL_27864_c0_g1_i2	–2.76	2.16 × 10^–2^	–5.98	1.26 × 10^–3^
Probable nuclear hormone receptor HR3 isoform X2	IIL_27960_c0_g1_i4	–5.04	9.87 × 10^–4^	–9.23	3.47 × 10^–5^
Steroid receptor partial	IIL_413_c0_g1_i1	–2.32	3.49 × 10^–2^	–3.46	1.33 × 10^–2^
Gonadotropin-releasing hormone receptor	IIL_42622_c0_g1_i1	4.75	7.69 × 10^–3^	5.64	6.02 × 10^–3^
Nuclear hormone receptor FTZ-F1 isoform X2	IIL_34249_c0_g1_i2	−	–	–8.54	8.85 × 10^–4^
Neuropeptide receptor A13	IIL_28896_c0_g1_i1	4.88	4.22 × 10^–2^	−	–
FMRFamide receptor-like	IIL_32921_c0_g1_i1	–2.79	1.47 × 10^–3^	−	–
Gonadotropin-releasing hormone receptor II	IIL_32102_c1_g6_i1	2.95	1.99 × 10^–3^	−	–
β-2-octopamine receptor	IIL_16542_c0_g1_i2	8.20	1.51 × 10^–4^	8.41	6.39 × 10^–4^

^*1^ - fold change values; ^*2–FDR^ - statistic values.

### Regulation of Cuticle Proteins and Peritrophic Membrane

Larval parasitization by *C. flavipes* also regulated larval cuticle formation and pigmentation. More than 100 cuticle proteins with RR-1 and RR-2 motifs, and larval and pupal cuticle proteins were inhibited in parasitized and pseudoparasitized larvae. Twenty-three cuticle proteins were down-regulated only in parasitized larvae and 51 only in pseudoparasitized larvae. Genes coding for enzymes involved in cuticle sclerotization and melanization, such as peroxidase isoform X1 (IIL_32355_c3_g1_i9: logFC = –8.75 in P; logFC = –8.78 in PP) and laccase 2 precursor (IIL_33503_c0_g1_i1: logFC = –2.93 in P; logFC = –3.75 in PP) were also down-regulated.

The peritrophic matrix lining the larval midgut of *D. saccharalis* was negatively affected by parasitization, through inhibition of peritrophin-1 (IIL_31190_c0_g1_i4: logFC = −2.72 in P; logFC = −3.11 in PP) and peritrophin precursor (IIL_32010_c0_g1_i1: logFC = −2.98 in P; logFC = −3.22 in PP), both of which are protein components of the peritrophic matrix.

### Regulation of Cellular Organization and Motility

Forty-three (43) genes involved in cellular organization and motility were down-regulated, while 35 were up-regulated in parasitized larvae. The transcripts filamin A isoform X1 (IIL_34274_c0_g2_i5: logFC = –10.69), dynactin subunit 5 (IIL_28346_c0_g1_i1: logFC = –10.91) and myosin heavy chain isoform X34 (IIL_33425_c0_g2_i10: logFC = −12.54) had their expression reduced over 1,000-fold in parasitized larvae. Genes involved in the inhibition of actin reorganization (Saarikangas et al., 2010), G-coupled protein Mth-3 receptor isoform X4 (IIL_31157_c3_g1_i3: logFC = 8) and twinfilin factor (IIL_34395_c1_g2_i3: logFC = 8.26), were strongly up-regulated in parasitized larvae. Other important genes involved in the regulation of cell adhesion and spread, α-tubulins, rho GTPase activators, and integrins, were represented by several isoforms in the transcriptome; a clear pattern of regulation could not be detected, as some isoforms were up- while others were down-regulated.

Pseudoparasitization by *C. flavipes* altered the expression of 81 transcripts of *D. saccharalis* larvae related to cellular cytoskeleton organization for cell motility and adhesion (down-regulated = 53, up-regulated = 28). In addition to the observed regulation of actin and myosin transcripts in parasitized larvae, down-regulation of signal activators of the transduction pathways that result in cell motility (Blanchoin et al., 2014), i.e., formin (IIL_28739_c0_g2_i2: logFC = –3.55), cdc42 factor (IIL_33959_c2_g1_i2: logFC = −10.01), and rho GTPase 6 activator isoform X2 (IIL_25884_c0_g1_i2: logFC = –3.41), was also observed in pseudoparasitized larvae.

The reorganization of the cytoskeleton for cell motility depends on calcium (Ca^+2^) signaling (Pinto et al., 2015; Tsai et al., 2015), and Ca^+2^ channels were regulated in parasitized larvae. The plasma membrane Ca^+2^-transporting ATPase 2 and Ca^+2^/Na^+1^ exchanger 1 isoform X1, which transport calcium to the extracellular space, were down-regulated (Table 2), but the influx Ca^+2^ channels ionotropic glutamate receptor, glutamate receptor kainate 2-like, and glutamate *N*-methyl-D-aspartate (NMDA) receptor associated 1 were up-regulated (Table 2). Phospholipase A2 and sphingosine 1-phosphate lyase were also down-regulated in parasitized larvae. These two proteins are channel regulators and they interfere with the levels of secondary messengers, through activation of G-protein (Table 2). The same pattern of regulation of influx and efflux Ca^+2^ channels was observed in pseudoparasitized larvae (Table 2). Genes encoding the Ca^+2^-modulated protein calmodulin and the Ca^+2^-activated proteins calcineurin A and B were up-regulated only in pseudoparasized larvae. No difference in their expression profile was detected in parasitized larvae (Table 2).

**TABLE 2:** Putative identification and fold-change values of sequences related to Calcium signaling pathway in larvae of *Diatraea saccharalis* parasitized (P) and pseudoparasitized (PP) by *Cotesia flavipes.*

**Putative identification**	**Transcripts**	**LogFC^∗^1 in P**	**FDR^∗^2**	**LogFC in PP**	**FDR**
Channels					
Ca^+2^ uptake mitochondrial isoforma X1	IIL_34181_c0_g1_i8	8.89	4.26 × 10^–5^	8.62	5.53 × 10^–4^
Plasma membrane Ca^+2^ transporting ATPase 2	IIL_33345_c1_g1_i12	–2.25	2.49 × 10^–2^	−	–
	IIL_33345_c1_g1_i4	–2.33	2.33 × 10^–3^	−	–
	IIL_33345_c1_g1_i5	–2.59	2.42 × 10^–2^	–3.13	1.84 × 10^–3^
Ca^+2^ homeostasis endoplasmatic reticulum isoforma X1	IIL_33424_c0_g1_i9	−	–	9.15	1.39 × 10^–4^
Canal de K + interno ATP-sensível 12	IIL_32994_c0_g1_i1	–2.88	2.23 × 10^–2^	−	–
Voltage-dependent L-type Ca^+2^ channel subunit β-2	IIL_34061_c0_g1_i1	–2.59	1.03 × 10^–3^	–2.27	1.41 × 10^–2^
	IIL_29702_c0_g1_i16	–2.74	1.18 × 10^–2^	–4.54	3.29 × 10^–3^
Sphingosine 1-phosphate lyase	IIL_32913_c0_g1_i3	10.21	3.26 × 10^–7^	11.18	5.81 × 10^–9^
Glutamate receptor kainate 2-like	IIL_31765_c1_g1_i2	2.91	3.95 × 10^–3^	−	–
	IIL_30235_c0_g1_i1	–3.23	2.07 × 10^–4^	–2.17	1.94 × 10^–2^
Glutamate *N*-methyl-D-aspartate receptor associated 1	IIL_31082_c0_g2_i5	8.49	4.74 × 10^–5^	9.99	7.18 × 10^–6^
	IIL_31765_c1_g1_i4	3.09	2.55 × 10^–2^	3.44	3.40 × 10^–2^
Inositol 1,4,5-trisphosphate receptor	IIL_33132_c1_g2_i2	–2.42	2.82 × 10^–2^	−	–
Ryanodine receptor	IIL_34501_c1_g1_i4	−	–	–2.35	2.21 × 10^–2^
Na^+^/CA^+2^ exchanger 1 isoform X1	IIL_23004_c0_g1_i1	–2.44	1.61 × 10^–2^	−	–
Carrier					
Ca^+2^ and integrin-binding 1-like	IIL_28526_c0_g1_i1	8.99	1.41 × 10^–5^	9.44	3.68 × 10^–5^
Ca^+2^ binding E63-1	IIL_31127_c4_g2_i21	−	–	8.48	1.08 × 10^–2^
	IIL_31127_c4_g2_i5	10.21	1.19 × 10^–4^	11.40	5.36 × 10^–11^
	IIL_31127_c4_g2_i17	−	–	–3.19	1.95 × 10^–3^
	IIL_31127_c4_g2_i20	4.02	1.19 × 10^–2^	−	–
	IIL_31127_c4_g2_i22	8.88	9.49 × 10^–9^	8.21	1.73 × 10^–6^
	IIL_31127_c4_g2_i9	5.96	3.46 × 10^–5^	5.25	6.72 × 10^–3^
	IIL_31127_c4_g2_i1	4.79	1.53 × 10^–3^	5.18	6.35 × 10^–3^
	IIL_31127_c4_g2_i14	–4.49	4.72 × 10^–5^	–3.93	1.65 × 10^–4^
	IIL_31127_c4_g2_i7	10.57	1.34 × 10^–7^	10.45	9.84 × 10^–7^
Sarcoplasmatic Ca^+2^ binding 1 isoform X2	IIL_29360_c0_g1_i2	−	–	8.36	4.41 × 10^–4^
FK506 binding	IIL_28237_c0_g1_i4	−	–	–4.61	6.74 × 10^–3^
	IIL_30991_c0_g1_i2	9.22	2.92 × 10^–6^	8.65	2.34 × 10^–4^
	IIL_31707_c0_g1_i4	3.80	1.80 × 10^–2^	3.82	4.41 × 10^–2^
Direct response					
Calcineurin A	IIL_23753_c0_g1_i1	9.80	1.25 × 10^–7^	9.38	2.38 × 10^–3^
Calcineurin B	IIL_31789_c3_g1_i2	−	–	9.18	3.38 × 10^–3^
	IIL_31789_c3_g1_i3	−	–	8.59	8.22 × 10^–3^
Calcyphosin isoform X3	IIL_29400_c0_g1_i4	–11.35	5.95 × 10^–11^	−	–
Calmodulin	IIL_31731_c1_g2_i5	−	–	7.25	2.72 × 10^–2^
	IIL_31846_c1_g2_i6	−	–	–3.36	7.59 × 10^–3^
Calpain-B-like isoform X3	IIL_29647_c2_g3_i9	10.16	1.65 × 10^–8^	10.29	3.64 × 10^–7^
Calphotin-like	IIL_27019_c0_g1_i1	–3.10	3.30 × 10^–4^	–3.63	6.03 × 10^–4^
EF-hand Ca^+2^ binding domain-containing 2	IIL_30305_c1_g1_i32	−	–	7.50	2.28 × 10^–2^
Phospholipase A2	IIL_31392_c0_g1_i8	6.27	1.05 × 10^–4^	−	–

^*1^^–^ fold change values in log format; ^*2– FDR^^–^ statistic values.

### Regulation of the Immune System

**FIGURE 7:**
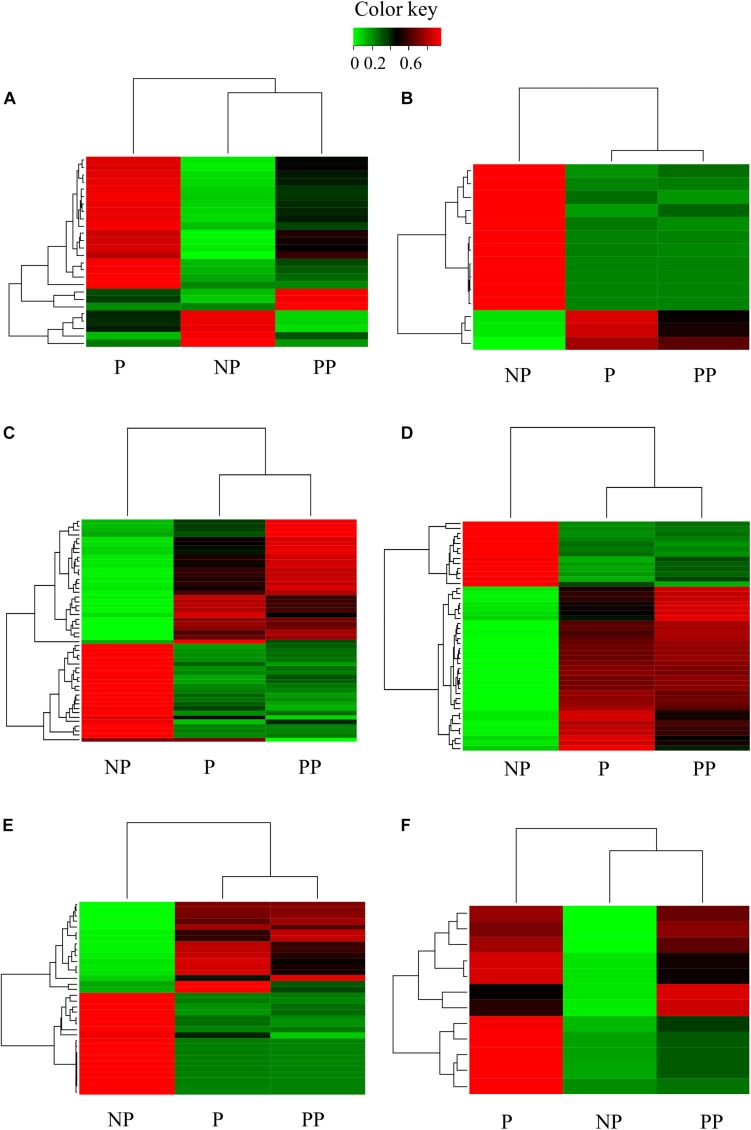
Heatmap of immune response transcripts of larvae of *Diatraea saccharalis* parasitized (P) and pseudoparasitized (PP) by *Cotesia flavipes*: **(A)** AMPs synthesis; **(B)** cellular response; **(C)** immune system activation; **(D)** immune system inactivation; **(E)** peptidoglycan recognition; **(F)** phenoloxidase enzymes.

*Cotesia flavipes* parasitizing larvae of *D. saccharalis* also regulated many transcripts involved with the immune system. We detected differential expression of 155 transcripts in parasitized larvae and 130 transcripts in pseudoparasitized larvae, most of them common to both conditions (Figure 7). In parasitized larvae, the wasps induced the synthesis of 19 antimicrobial peptides (AMPs), of which attacin, cecropin, scolexin and gloverin were highly expressed (Figure 7A). In pseudoparasitized larvae, only nine differentially expressed AMPs were detected. Expression of cecropin (IIL_23051_c0_g1_i2: logFC = 2.55 in P) and defensin (IIL_25072_c0_g1_i1: logFC = 12.74 in P) was not altered in pseudoparasitized larvae. AMP synthesis in insects is regulated by the *Imd* (*immune deficiency*) and *Toll* pathways of the immune system (De Gregorio et al., 2002), and Toll-like proteins (IIL_34261_c0_g1_i8: logFC = 2.59 in P; IIL_34261_c0_g1_i10: logFC = 2.05 in P; IIL_34261_c0_g1_i11: logFC = 2.59 in P; logFC = 2.55 in PP; IIL_34261_c0_g1_i4: logFC = 4.85 in P; logFC = 5.20 in PP; IIL_34261_c0_g1_i7: logFC = 6.79 in P; logFC = 7.36 in PP) and Toll receptors (IIL_28993_c0_g1_i1: logFC = 4.94 in PP) were up-regulated in both parasitized and pseudoparasitized larvae.

*Cotesia flavipes* also affected a group of pattern-recognition peptides involved in the humoral response of *D. saccharalis* larvae. Peptidoglycan-recognition peptide, lipopolysaccharide binding, and lipopolysaccharide-induced tumor necrosis factor-α were up-regulated in parasitized and pseudoparasitized larvae (Figure 7E).

Contrary to the activation of the humoral immune response through the stimulation of AMPs expression, genes involved in activation of the cellular immune response were down-regulated in larvae of *D. saccharalis* parasitized and pseudoparasitized by *C. flavipes* (Figure 7B). Sequences of macrophage mannose receptor 1-like, c-type lectin (IIL_33289_c2_g8_i1: logFC = –2.06 in P; logFC = –2.17 in PP; IIL_31980_c2_g1_i1: logFC = –5.26 in P; logFC = –11.49 in PP), immunolectin (2a and 4) and hemolin (IIL_31301_c0_g1_i1: logFC = –7.49 in P; logFC = –7.70 in PP; IIL_31301_c0_g1_i2: logFC = –8.12 in P; logFC = –7.88 in PP) were all inhibited in parasitized and pseudoparasitized hosts (Figure 7B).

Phenoloxidases that participate in both the humoral and cellular immune response were severely regulated in parasitized and pseudoparasitized larvae, through inhibition of the expression of serine proteases (Figure 7C) that activate the prophenoloxidase cascade, and stimulation of the expression of 16 serine protease inhibitors (serpins) (Figure 7D). All of the serine proteases analyzed in this case carried the clip-domain typical of proteases that function as immune factors (Kanost and Jiang, 2015). Twelve additional serpin transcripts were up-regulated only in parasitized larvae, while four other serpin transcripts were up-regulated exclusively in pseudoparasitized larvae. Up-regulation of the NF-κ-β inhibitor cactus (IIL_31869_c2_g1_i4: logFC = 3.19; IIL_31869_c2_g1_i6: logFC = 2.43) was detected only in parasitized larvae, while down-regulation of the nuclear factor NF-κ-β p110 subunit (IIL_30756_c0_g3_i8: logFC = –2.26) was detected only in pseudoparasitized larvae. The expression of a particular set of isoforms of phenoloxidase subunits 1 and 2 was affected differently in parasitized and pseudoparasitized larvae of *D. saccharalis.* PO 1 and 2 were up-regulated in parasitized larvae, but the expression of these transcripts in pseudoparasitized larvae was similar to that observed in non-parasitized larvae (Figure 7F).

### Regulation of Host Metabolism

*Cotesia flavipes* also targeted genes involved in nutrient metabolism and transport. Several transcripts involved in carbohydrate metabolism were up-regulated in parasitized larvae, particularly those from the glycolysis and gluconeogenesis pathways. Phosphoglucose isomerase (IIL_34193_c0_g2_i3: logFC = 3.48), phosphoglucomutase (IIL_34190_c0_g1_i5: logFC = 2.69), aldose 1-epimerase-like (IIL_31340_c4_g3_i2: logFC = 3.15 in P; logFC = 3.48 in PP), and fructose-1,6-bisphosphatase (IIL_30548_c2_g3_i1: logFC = 7.62) were up-regulated, while only two were down-regulated. Hexokinase-2-like isoform X2 (glycolysis) (IIL_33352_c0_g3_i1: logFC = –2.64 in P; logFC = −2.51 in PP) and enolase (gluconeogenesis) (IIL_28113_c0_g1_i1: logFC = –8.78 in P; logFC = –9.51 in PP) were both inhibited in parasitized and pseudoparasitized larvae. The trehalose pathway was also affected in larvae parasitized and pseudoparasitized by *C. flavipes*, with the up-regulation of trehalose-6-phosphate synthase (IIL_32431_c0_g1_i3: logFC = 2.07 in P; IIL_32431_c0_g1_i6: logFC = 11.58 in P, logFC = 11.79 in PP).

The carrier protein apolipo-D (IIL_29978_c0_g3_i3: logFC = –2 in P; IIL_29978_c0_g3_i6: logFC = –4.78 in P; logFC = –9.66 in PP) and long-chain fatty acid transport 4-like (IIL_31821_c1_g1_i11: logFC = –6.06 in P; logFC = 4.69 in PP; IIL_31821_c1_g1_i14: logFC = –4.07 in P; logFC = –4.83 in PP) were down-regulated in parasitized and pseudoparasitized larvae. Sugar and trehalose transporters were represented by a number of isoforms with no clear pattern of regulation, as some were up- and others were down-regulated.

Regulation of nutrient metabolism and transport was followed by regulation of lipid and protein storage by *C. flavipes*. The lipid storage droplets surface-binding 1 isoform X1 (IIL_34294_c2_g1_i2: logFC = 2.89 in P; logFC = 2.17 in PP; IIL_34294_c2_g1_i4: logFC = 10.39 in P; logFC = 10.18 in PP), arylphorin subunit α (IIL_28532_c2_g2_i4: logFC = 4.45 in P; logFC = 4.20 in PP) and acidic juvenile hormone-suppressible 1-like protein (IIL_DN34340_c0_g1_i6: logFC = 2.79 in P) were up-regulated.

## Discussion

Differential gene expression showed that *C. flavipes* interferes with the expression of 2,459 transcripts (5%) of *D. saccharalis*, revealing the large number of target genes involved in many physiological processes that are essential for the successful growth and development of the parasitoid. A similar number of transcripts (2,225) were regulated in larvae of *D. saccharalis* that were pseudoparasitized by *C. flavipes*. The similar numbers of differentially expressed genes in parasitized and pseudoparasitized larvae of *D. saccharalis* indicate that most of the virulence factors required for host regulation by *C. flavipes* are injected by the female wasp during parasitization, and would include venom, ovarian proteins and viral particles.

The role of venom and PDVs in the process of host regulation has been demonstrated by injecting them individually into the hosts of parasitoids, including a number of host-parasitoid associations involving braconids, such as *Chelonus inanitus* (L.) *– Spodoptera littoralis* (Boisduval) (Lepidoptera: Noctuidae) (Hochuli et al., 1999), *Protapanteles liparidis* (Bouché) *– Lymantria dispar* (L.) (Lepidoptera: Erebidae) (Hoch et al., 2009), *Cotesia plutellae* (Kurdjummov) *– Plutella xylostella* (L.) (Lepidoptera: Plutellidae) (Yu et al., 2007), and the system studied here, *C. flavipes – D. saccharalis* (Lopes, 2008). The synthesis and release of molecules by teratocytes (Nakamatsu et al., 2002) and the anal vesicle of the larval parasitoid and their participation in host regulation have been demonstrated in several host-parasitoid associations (Kaeslin et al., 2006). Our data showed that the release of molecules by the developing parasitoid larvae and/or teratocytes also contributes to host regulation. However, the contribution of virulence factors from teratocytes and/or larval secretions does not have a strong impact on gene expression, at least in the host genes we identified in this study.

### Regulation of Cell Cycle and Apoptosis

Activation of secondary messengers is required to trigger signal transduction and allow cells to perceive changes in the surrounding environment (Clapham, 2007). Calcium (Ca^+2^) ions are among the most important transduction-signal mediators, affecting cell excitability, exocytosis, motility, apoptosis and transcription (van Haasteren et al., 1999; Bernstein, 2015; Humeau et al., 2017). The increase in intracellular levels of Ca^+2^ is perhaps the most universal signaling cascade required for cell proliferation (Pinto et al., 2015). Ca^+2^ signaling was strongly regulated in larvae of *D. saccharalis* parasitized and pseudoparasitized by *C. flavipes*. *Cotesia flavipes* inhibited the expression of Ca^+2^ efflux channels and stimulated Ca^+2^ influx channels. Muscle contraction requires the release of calcium from the sarcoplasmic reticulum to the muscle sarcolema to promote the disassociation of the troponin–tropomyosin complex from the binding sites of the actin filament, allowing myosin to bind to actin for cell contraction. Alteration on muscle contraction can be related to change of host behavior induced by parasitoids, as *Glyptapanteles* sp. that induces their caterpillar host to behave as a bodyguard of the parasitoid pupae and knocks off predators with violent head-swings (Grosman et al., 2008). An increase in calcium availability reduces cell adhesion and activates caspases involved in cytolysis and DNA degradation during apoptosis. Calcium regulation can also lead to a disruption in the cell cycle (Mattson and Chan, 2003; Devreotes and Horwitz, 2015; Humeau et al., 2017).

Some caspases and Ca^+2^-dependent molecules (caspase activators), such as calpain and calcineurin, were up-regulated in *D. saccharalis* larvae parasitized and pseudoparasitized by *C. flavipes* (Mattson and Chan, 2003; Humeau et al., 2017). Apoptosis of *High Five* cells was induced in the presence of viral protein associated with *Microplitis bicoloratus* Chen (Hymenptera: Braconidae) and *Toxoneuron nigriceps* (Viereck) (Hymenoptera: Braconidae), through their interaction with cell proteins that activate caspases (Yu et al., 2016; Salvia et al., 2017). *Cotesia flavipes* also regulated Ca^+2^ channels, Ca^+2^ modulators and Ca^+2^-dependent molecules, altering several physiological processes ranging from cell proliferation to apoptosis activation.

### Hormonal Regulation

Targeting of the host endocrine system for regulation is well described for several host-parasitoid systems. In the *D. saccharalis* – *C. flavipes* system, we observed intensive regulation of synthesis and degradation of juvenile hormone (JH) and ecdysteroids. *Cotesia flavipes* induced synthesis of juvenile hormone in both parasitized and pseudoparasitized larvae. Regulation of JH by *C. flavipes* is obtained by virulence factors injected with the eggs, and the same effect was observed in larvae in which virulence factors from teratocytes and parasitoid larvae were not available (pseudoparasitized larvae). JH regulation is important to maintain the parasitized host at the larval stage as long as is necessary for the parasitoid larva to fully develop (Lawrence, 1986; Grossniklaus-Bürgin et al., 1998; Noriega, 2014), but also to regulate the expression of juvenile hormone-dependent genes, such as methionine-rich protein (Salvador and Cônsoli, 2008), and the allocation of nutrients to host growth and development (Jones, 1995; Cônsoli and Vinson, 2012). Contrariwise, the juvenile hormone-degrading enzymes esterase and epoxide hydrolase were also up-regulated. However, up-regulation of genes involved in JH synthesis was much more pronounced than the up-regulation seen in JH-degrading enzymes. Additionally, we cannot rule out the possibility that the up-regulation of the translational regulatory factors observed in parasitized and pseudoparasitized larvae of *D. saccharalis* could be interfering with the synthesis of enzymes involved in the synthesis and degradation of JH. A multiomic approach would be required for a full understanding of the functional regulation of JH titers in parasitized larvae. However, JH regulation varies widely among host-parasitoid systems and often depends on the strategy of parasitoid development. Endoparasitoids such as *G. liparidis* and *Tranosema rostrale* (Brischke) (Hymenoptera: Ichneumonidae), maintain elevated JH titers by reducing the host JH esterases (Béliveau et al., 2000; Schafellner et al., 2007). The larval parasitoid *T. nigriceps* does not interfere with the JH esterase levels in the host, but regulates other components of the hormone pathways (Li et al., 2003). On the other hand, in egg-pupal or larval-pupal parasitoids that complete their development in the host pupal stage, such as *C. inanitus*, JH esterase levels are elevated to induce reduction in JH titers in order to induce a precocious onset of the pupal stage in the host *S. littoralis* (Kaeslin et al., 2005).

*Cotesia flavipes* parasitization of *D. saccharalis* larvae led to the down-regulation of enzymes acting on ecdysteroid synthesis and of several eukaryotic translation initiation factors. Eukaryotic translation initiation factors also participate in regulating the activity of prothoracic glands as signaling factors (Gu et al., 2017). Down-regulation of ecdysteroid synthesis was followed by the negative regulation of many orphan nuclear receptors, such as the nuclear hormone receptors HR3, E75 and E74. Activation of orphan nuclear receptors is dependent on ecdysteroid induction. E75 responds faster to ecdysteroid activation than HR3, and both of these are also considered transcription factors by some authors (Hannas and LeBlanc, 2010). These orphan receptors heterodimerize to act as fundamental regulators of changes in development and redirection of metamorphosis induced by ecdysteroids. The HR3 receptor orchestrates the transition from larva to prepupa and the E75 receptor modulates this transition (Thummel, 2005; Li K. et al., 2015). Disruption of the expression of the E75 and HR3 receptors will affect the onset of metamorphosis, as they are in charge of activating and deactivating genes in preparation for pupation, in response to the first ecdysteroid peak (Horner et al., 1995; Lam et al., 1997; Guo et al., 2015). In addition to inhibiting the E75 and HR3 receptors to control cell response to ecdysteroid stimulation, *C. flavipes* induced the overexpression of ecdysone oxidase and CYP P450 18A1, which are ecdysteroid-inactivating enzymes (Guittard et al., 2011; Li Z. et al., 2015). Thus, ecdysteroid availability and cell recognition are negatively affected in parasitized and pseudoparasitized larvae, demonstrating that cells would be unresponsive to ecdysteroid stimulation even if hormone synthesis was not affected.

Down-regulation of ecdysteroid titers will block important pathways for host ecdysis such as chitin metabolism and cuticle degradation and synthesis (Charles, 2010). Thus, the strong down-regulation of cuticle proteins, chitinases and chitin-synthesis proteins in parasitized and pseudoparasitized larvae is a consequence of the regulation of genes involved in ecdysteroid synthesis, as observed in *D. saccharalis* larvae parasitized by *C. flavipes*.

### Regulation of Cellular Organization and Motility

Bracoviruses are reported to interfere with host cell activities, mainly disrupting the cell cytoskeleton by expressing proteins that irreversibly bind to gene-expression factors (Suderman et al., 2008; Bitra et al., 2012). Several genes that are important to the cell cytoskeleton (actin and myosin) and to cell migration and attachment (integrin) were regulated in larvae of *D. saccharalis* parasitized and pseudoparasitized by *C. flavipes*. Regulation of these genes interferes with the ability of hemocytes to move, attach to and spread over the surface of the invader egg during egg encapsulation (Webb and Luckhart, 1994; Kwon and Kim, 2008; Devreotes and Horwitz, 2015). Disruption of the cell cytoskeleton also affects the cell activity in the host. PDV viral ankyrins interfere with the cytoskeleton organization of cells of the prothoracic gland, affecting the synthesis and transport of ecdysteroids (Valzania et al., 2014). Thus, regulation of genes that affect the cell cytoskeleton organization, cell motility, attachment and spread would be associated with virulence factors produced by the bracovirus associated with *C. flavipes*.

### Regulation of the Immune System

Regulation of the host immune responses is an important function of the virulence factors that female parasitic wasps inject into their hosts during parasitization. Host humoral and cellular immune responses are targeted by parasitic wasps to prevent the encapsulation process (Vinson, 1990; Schmidt et al., 2001; Barat-Houari et al., 2006; Mahmoud et al., 2011; Meng et al., 2016). Initial reports on *D. saccharalis – C. flavipes* demonstrated larval parasitization induced an increase in the population of host hemocytes and in the phenoloxidase activity in permissive and non-permissive hosts. Interestingly, increase in host hemocytes did not correlate with parasitoid encapsulation by the host (Alleyne and Wiedenmann, 2001; Mahmoud et al., 2012). A large number of genes of *D. saccharalis* were differentially regulated in larvae parasitized or pseudoparasitized by *C. flavipes.* Expression of C-type lectins and hemolins was severely affected in these parasitized or pseudoparasitized larvae. Regulation of the expression of these genes would affect pattern recognition of pathogens and cell adhesion, and the similarities in the expression profiles in parasitized and pseudoparasitized larvae show that the maternally associated virulence factors are responsible for altering the expression of these genes. Down-regulation of C-type lectins and hemolins indicates that the host immune response is compromised upon parasitization (Fang et al., 2011; Wang et al., 2012). Hemolin plays several roles in the immune response of insects. Hemolin is highly important to mount the cell response to bacterial infections (Sun et al., 1990; Schmidt et al., 1993; Lanz-Mendoza et al., 1996), but hemolin has also been shown to affect the humoral immune response by interfering with phenoloxidase activation (Terenius et al., 2007). Additionally, hemolin has been suggested to be important in the immune response against viruses attacking lepidopterans (Terenius, 2008; Qian et al., 2017), and the down-regulation observed in larvae of *D. saccharalis* parasitized and pseudoparasitized by *C. flavipes* is likely required to prevent the host from defending against the polydnavirus particles injected by female wasps. Inhibition of hemolin expression in larvae of *Manduca sexta* (L.) (Lepidoptera: Sphingidae) parasitized by *Cotesia congregata* (Say) (Hymenoptera: Braconidae) was shown to occur soon after the polydnavirus gene *Cc*V1 was expressed (Labropoulou et al., 2008).

Besides the possible regulation of phenoloxidases by hemolin, two other genes (serine proteases and serpins) belonging to the prophenoloxidase cascade (PPO) were regulated in larvae parasitized and pseudoparasitized by *C. flavipes.* Inhibition of the expression of serine proteases and overexpression of serine protease inhibitors (serpins) (Irving et al., 2000; Gorman and Paskewitz, 2001) show that inhibiting the activation of phenoloxidase is important for suppressing the immune response of the host against *C. flavipes.* PPO and phenoloxidases bind to foreign and hemocyte surfaces and participate in the process of hemocyte melanization and encapsulation of large pathogens and parasitoids (Ling and Yu, 2005).

Regulation of the immune system of *D. saccharalis* also involved controlling the expression of a key member of the immune response, the nuclear factor kappa-light-chain-enhancer of activated B cells (NF-κ-β). NF-κ-β is a complex of proteins that once activated, controls DNA transcription, cell survival and cytokine production and participates in several cell responses, including the response to infections (Perkins, 2007). *Cotesia flavipes* inhibited the activity of NF-κ-β in parasitized and pseudoparasitized larvae of *D. saccharalis* by inducing the overexpression of cactus, an NF-κ-β inhibitory factor (Shrestha et al., 2009). Regulation of NF-κ-β then leads to a change in cell function, impacting hematopoietic and immune signaling. Gueguen et al. (2013) proposed that regulation of NF-κ-β by PDV vankyrins leads to an immune deficiency that contributes to the successful colonization of the host and survival of the developing parasitoid larvae. The similar profile of gene expression in larvae of *D. saccharalis* parasitized and pseudoparasitized by *C. flavipes* demonstrates that the regulation of the host immune system seems to rely exclusively on maternal virulence factors.

Regulation of the host’s immune response is certainly important for successful parasitization, but parasitoids also must maintain their host’s immune responses active up to a certain level, in order to provide a healthy environment to support their own development. For this reason, antimicrobial peptides (AMPs) are not always inhibited in parasitized hosts. Up-regulation of AMPs was observed in larvae of *D. saccharalis* parasitized and pseudoparasitized by *C. flavipes*, although the levels of regulation were much higher in parasitized than in pseudoparasitized larvae, indicating that other virulence factors or late factors of regulation are required to maintain the high levels of expression observed in parasitized larvae. Activation of AMP expression requires activation of the *Toll* and *Imd* pathways (De Gregorio et al., 2002; Myllymaki et al., 2014), and several Toll-like proteins were up-regulated by *C. flavipes*.

### Regulation of Host Metabolism

Modulation of the host’s energy metabolism will vary according to the strategy of parasitoid development, and is usually accompanied by manipulation of the host’s endocrine system. Although glycolysis and gluconeogenesis have opposing functions, they share several enzymes that play reversible roles in each pathway (Thompson, 2003). These pathways are regulated by the activation of insulin-like peptides in response to the host’s nutritional environment (Mirth and Riddiford, 2007). The sugar content in the hemolymph of *D. saccharalis* larvae parasitized by *C. flavipes* is maintained almost unaltered throughout parasitoid development (Salvador and Cônsoli, 2008; Rossi et al., 2014). Maintenance of sugar levels in the hemolymph of larvae of *M. sexta* parasitized by *C. congregata* has been found to occur through increased gluconeogenesis and trehalose synthesis (Thompson and Dahlman, 1999; Thompson, 2001). The expression levels of trehalose-6-synthase were elevated in parasitized and pseudoparasitized larvae of *D. saccharalis*, but activation of gluconeogenesis in parasitized/pseudoparasitized larvae is not fully understood. *Cotesia flavipes* inhibited the initial steps of gluconeogenesis (enolase down-regulation) while activating the final steps (up-regulation of phosphoglucose isomerase, phosphoglucomutase, fructose-1,6-bisphosphatase, and aldose 1-epimerase-like). Although our data suggest that regulation of the sugar content in the host is a means to make this nutrient available to the developing parasitoid, we cannot discard the possibility that some of the intermediates also participate in other physiological processes. For example, enolases can act as DNA-binding proteins and affect gene transcription or participate in muscle growth and development, among other processes (Ji et al., 2016). Contrary to the inhibition of enolase observed in *D. saccharalis* parasitized by *C. flavipes*, the aphid parasitoid *Aphidius ervi* Haliday (Hymenoptera: Braconidae) increases the availability of enolase in the host, releasing enolases produced by teratocytes (Falabella et al., 2009).

Similarly, the positive regulation of the expression of host proteins and enzymes involved in lipid synthesis could be a strategy of the parasitoid to maintain a suitable nutritional environment for its development. Nevertheless, because *C. flavipes* regulates several translational regulatory factors, it is difficult to understand the outcome of regulating any of these components individually (Kim, 2007).

## Conclusion

We identified several pathways and key genes of the host *D. saccharalis* that are regulated by the wasp *C. flavipes*, using a *de novo* assembly of a transcriptome of this non-model species. We conclude that most of the regulation of gene expression of *D. saccharalis* is controlled by virulence factors transmitted by the female wasp along with the egg, as the gene-expression profile in parasitized larvae was similar to that in pseudoparasitized larvae. Our undesired need to pool RNA samples at different sampling time after host parasitization in order to build one sequencing library frustrated our expectations to clearly identify and differentiated early and late target genes of the virulence factors of *C. flavipes*. Nevertheless, we depicted the efficiency of the virulence factors of *C. flavipes* in regulating gene expression of *D. saccharalis*, and propose that *C. flavipes* regulates a large number of physiological processes of *D. saccharalis* by targeting mechanisms that control the availability of calcium ions, due to the importance of this molecule in almost every aspect of the cell life cycle. Finally, our data provide an overview of the changes induced in the host by *C. flavipes*, generating a large body of information for future functional studies.

## Author Contributions

BM and FC designed and planned the research, and revised and edited the final manuscript. FC secured the funds, supervised the data analysis, and revised, edited, and amended the initial draft of the manuscript. BM collected and analyzed the data, and wrote the initial draft of the manuscript.

## Conflict of Interest Statement

The authors declare that the research was conducted in the absence of any commercial or financial relationships that could be construed as a potential conflict of interest.
