# Supplementary material for: Regulation of the Larval Transcriptome of Diatraea saccharalis (Lepidoptera: Crambidae) by Maternal and Other Factors of the Parasitoid Cotesia flavipes (Hymenoptera: Braconidae)
Source: Front Physiol. 2019 Sep 6;10:1106. doi: 10.3389/fphys.2019.01106 (PMC6742964; doi:10.3389/fphys.2019.01106)
Supplement: Supplementary file 1 [file Table_1.DOCX]

Frontiers in Physiology – On line Supplementary Material

Regulation of the larval transcriptome of *Diatraea saccharalis* (F.) (Lepidoptera: Crambidae) by maternal and larval factors of the parasitoid *Cotesia flavipes* Cameron (Hymenoptera: Braconidae)

Bruna Laís Merlin, Fernando Luis Cônsoli

Insect Interactions Laboratory, Department of Entomology and Acarology, College of Agriculture “Luiz de Queiroz”, University of São Paulo, Piracicaba, SP, Brazil

**Table S1**. Number of reads obtained for each sample of control and larvae of *Diatraea saccharalis* parasitized (P) and pseudoparasitized (PP) by *Cotesa flavipes* after cDNA sequencing via Illumina © platform and quality analysis

| Sample | Replicate | | Number of eliminated reads | Number of unpaired reads | Number of paired reads |
| --- | --- | --- | --- | --- | --- |
| NP | 1 | 230,247 | | 729.,431 | 19,131,634 |
|  | 2 | 191,268 | | 418,293 | 18,150,972 |
|  | 3 | 209,872 | | 638,348 | 18,500,057 |
| P | 1 | 185,060 | | 680,438 | 20,386,766 |
|  | 2 | 204,351 | | 740,772 | 21,065,076 |
|  | 3 | 167,389 | | 613,546 | 18,227,198 |
| PP | 1 | 149,745 | | 530,288 | 16,953,358 |
|  | 2 | 173,561 | | 576,053 | 16,935,608 |
|  | 3 | 190,601 | | 661,880 | 19,869,640 |

**Table S2**. Statistic summary of *de novo* assembly of the larval transcriptome of *Diatraea saccharalis* from reads obtained in an Illumina© platform

|  | **Number** | **Mean size (bp)** | | **Total of bases** |
| --- | --- | --- | --- | --- |
| **Transcripts** | 144,116 | 861.55 | 1,244,163,268 | |
|  |  |  |  | |
| ***Assembly*** | **Size (bp)** |  |  | |
| **N_10_** | 3,866 |  |  | |
| **N_20_** | 2,836 |  |  | |
| **N_30_** | 2,233 |  |  | |
| **N_40_** | 1,800 |  |  | |
| **N_50_** | 1,445 |  |  | |


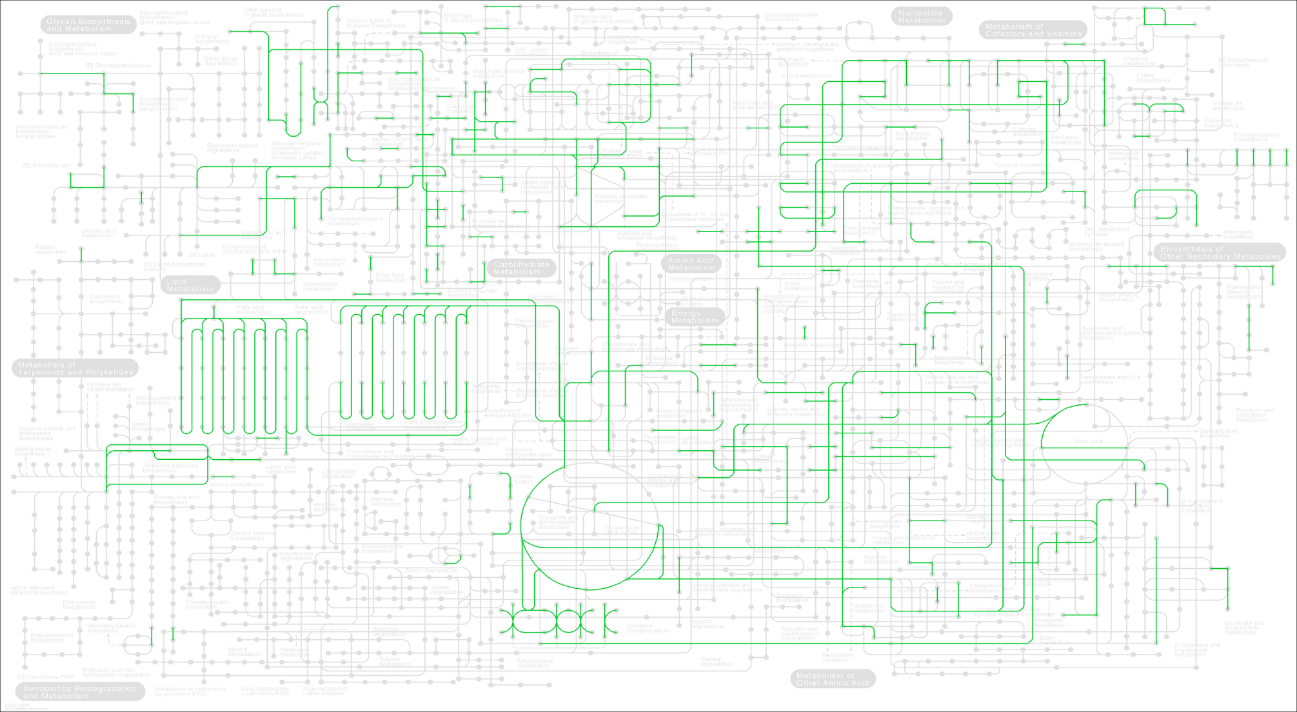


**Figure S1**. Global map of metabolic pathways deposited in KEGG databank. Pathways in green are represented in *de novo* assembly of *Diatraea saccharalis*
